# Identifying interactive biological pathways associated with reading disability

**DOI:** 10.1002/brb3.1735

**Published:** 2020-06-28

**Authors:** Hope Sparks Lancaster, Xiaonan Liu, Valentin Dinu, Jing Li

**Affiliations:** ^1^ College of Health Solutions Arizona State University Tempe AZ USA; ^2^ Department of Computing, Informatics, and Decision Systems Engineering Schools of Engineering Arizona State University Tempe AZ USA; ^3^ School of Industrial and Systems Engineering Georgia Institute of Technology Atlanta GA USA

**Keywords:** cognition, development, genetics, informatics

## Abstract

**Introduction:**

Past research has suggested that reading disability is a complex disorder involving genetic and environment contributions, as well as gene–gene and gene–environment interaction, but to date little is known about the underlying mechanisms.

**Method:**

Using the Avon Longitudinal Study of Parents and Children, we assessed the contributions of genetic, demographic, and environmental variables on case–control status using machine learning. We investigated the functional interactions between genes using pathway and network analysis.

**Results:**

Our results support a systems approach to studying the etiology of reading disability with many genes (e.g., *RAPGEF2*, *KIAA0319*, *DLC1*) and biological pathways (e.g., neuron migration, positive regulation of dendrite regulation, nervous system development) interacting with each other. We found that single nucleotide variants within genes often had opposite effects and that enriched biological pathways were mediated by neuron migration. We also identified behavioral (i.e., receptive language, nonverbal intelligence, and vocabulary), demographic (i.e., mother's highest education), and environmental (i.e., birthweight) factors that influenced case–control status when accounting for genetic information.

**Discussion:**

The behavioral and demographic factors were suggested to be protective against reading disability status, while birthweight conveyed risk. We provided supporting evidence that reading disability has a complex biological and environmental etiology and that there may be a shared genetic and neurobiological architecture for reading (dis)ability.

## Graphical abstract

We completed a pilot analysis using elastic net regression and pathway analysis. Our pilot results highlight the role of brain development and neuron organization biological pathways in reading disability status.

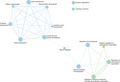

## 1 INTRODUCTION

Reading disability is a heritable neurodevelopmental condition with a typical age of identification between 7 and 10 years old and affects about 10% of all school‐aged children (Kamhi & Catts, 2012). Reading disability is a complex disorder affecting a number of skills and abilities, including problems with decoding (Catts, 2017), delayed and disordered phonological processing (Beitchman & Young, 1997; Clercq et al., 2017; Peter, Lancaster, Vose, Middleton, & Stoel‐Gammon, 2017), reduced language functioning (Tomblin, Zhang, Buckwalter, & Catts, 2000; Torppa, Lyytinen, Erskine, Eklund, & Lyytinen, 2010), potential deficits in working memory performance (Beneventi, Tonnessen, & Ersland, 2009; Cirino et al., 2018), and abnormal auditory and visual processing (Rendall, Perrino, LoTurco, & Fitch, 2019; Sharma, Purdy, & Kelly, 2009; Sperling, Lu, Manis, & Seidenberg, 2005). Additionally, individuals with reading disability process reading, language, auditory, and visual information differently compared to peers in neuroimaging studies (D'Mello & Gabrieli, 2018; Martin, Kronbichler, & Richlan, 2016; Martin, Schurz, Kronbichler, & Richlan, 2015). Combined the symptoms of reading disabilities negatively affect academic achievement during school‐age years (Daniel et al., 2006; Helland & Asbjornsen, 2003; Morken & Helland, 2013) and have lifelong impacts, such as reduced job attainment (Beer, Engels, Heerkens, & van der Klink, 2014) and increased risk of psychiatric difficulties (Daniel et al., 2006). Current theories hypothesize that the behavioral differences are the result of the neurological differences which seem to be driven by underlying genetic differences (Landi & Perdue, 2019; Mascheretti et al., 2017). Therefore, understanding the genetic and underlying biological etiologies of reading disability could improve theoretical and clinical models for diagnosing and treating reading disability.

Past research has established a genetic contribution for reading disability (Facoetti, Gori, Vicari, & Menghini, 2019; Gialluisi, Guadalupe, Francks, & Fisher, 2017; Landi & Perdue, 2019; Mascheretti et al., 2017; Paracchini, Diaz, & Stein, 2016; Skeide et al., 2015), starting with evidence that reading disability is highly heritable with estimates between 40 and 60 percent (Wadsworth, DeFries, Olson, & Willcutt, 2007; Willcutt et al., 2011). A substantial body of literature has investigated the complex genetic contributions for reading disability, considering single gene associations, gene–gene interactions, and gene–environment interactions. The major findings are briefly reviewed as follows. Multiple studies have associated nine candidate regions and 14 candidate genes with dyslexia, one type of reading disability (Gibson & Gruen, 2008; Newbury, Monaco, & Paracchini, 2014; Willcutt et al., 2011). These genes include the following: *DYX1C1*, *DCDC2*, *KIAA0319*, *C2ORF3*, *MRPL19*, *ROBO1*, *FAM176A*, *FMR1*, *S100B*, *DOCK4*, *KIAA0319L*, *DIP2A*, *GTF2I*, and *GRIN2B*. Research has suggested that reading disability is polygenic in nature and that these genes interact with each other and with environmental factors (Friend, DeFries, Wadsworth, & Olson, 2007; Gayán & Olson, 2001; Price et al., 2020). In the context of this research area, environmental factors refer to a broad set of mostly nongenetic predictors of reading disability status, such as biological sex, birthweight, gestational weeks, mother's highest education, and language ability (Mascheretti, Andreola, Scaini, & Sulpizio, 2018). Mascheretti, Bureau, Trezzi, Giorda, and Marino (2015) investigated gene–gene interactions in reading disability and found that *KIAA0319/TTRAP* and *DYX1C1* interact with *GRIN2B* for predicting performance on short‐term memory tasks in children with reading disability. There is evidence that the genes associated with reading disability interact with each other at the functional level, as many of these genes are involved in several brain development process. Past research has linked many of candidate genes to neuronal migration, neurite outgrowth, cortical morphogenesis, and ciliary structure and function (Newbury et al., 2014). Neuronal migration has been suggested as the neurological basis for dyslexia in prior studies (Martin et al., 2015, 2016). Mascheretti et al. (2018) performed a systematic review of studies examining environmental factors for dyslexia and reported birthweight and gestational weeks were predictive of dyslexia status and possible interplay between genetic risk and teacher quality and parental education. Recent research has provided some insights into how genes and environmental factors may interact (Gu et al., 2018; Kershner, 2019; Mascheretti et al., 2018; Mascheretti et al., 2013). Gu et al. (2018) examined the interaction between genetic variants in *CNTNAP2* and environmental factors and found sex specific interaction; specifically, they found that two variants (rs3779031 and rs987456) in CNTNAP2 were associated with reading disability status in females but not males and that the interaction between rs987456 and scheduled reading time was protective in females. In summary, the past research has revealed that the genetic contributions are a complex system with multiple genes involved, as well as gene–gene and gene–environment interactions.

Despite the recent advances in understanding the genetics of reading disability, there is still much that is not understood. Past genetic studies about reading disability are limited because the genetic associations were evaluated on a one‐gene‐at‐a‐time basis, which is inefficient for identifying genetic contributions in complex phenotypes due to statistical constraints and the inability to represent complex etiologies. Therefore, past research may have missed important genetic factors that contribute to or protect against reading disability. An additional complication is that environmental and demographic factors interact with genetic factors but a limited number of studies have examined gene–environment interactions (Becker et al., 2017; Gu et al., 2018; Jerrim, Vignoles, Lingam, & Friend, 2015). No studies to our knowledge have integrated genetic, environmental, and demographic data within the same analysis due to constraints imposed by research design and statistical analysis. Because our understanding of the genetics is limited by prior statistical constraints, we do not know how many genes are relevant for understanding the genetics of reading disability, which biological pathways are crucial, or how including environment and demographic factors influence genetic associations.

In this paper, we perform a novel study different from prior approaches by hypothesizing that there are (a) multiple genetic markers, environmental, and demographic factors involved in reading disability, (b) informative genetic markers are overrepresented within certain biological process pathways, and (c) genetic markers can be positively and negatively associated with reading disability status. Our approach is to take advantage of modern machine learning developments that provide effective and efficient approaches for big data modeling and analysis. Specifically, we use a sparse learning method called elastic net (Waldmann, Mészáros, Gredler, Fuerst, & Sölkner, 2013) to identify an array of genetic markers—single nucleotide polymorphisms (SNPs, also known as single nucleotide variants)—predictive of reading disability simultaneously. This approach overcomes the limitation of past research primarily dependent on multiple test correction applied to results of univariate analysis. The correction is known to be overly strict and thus having the risk of missing important genetic associations (Stein et al., 2012; Waldmann et al., 2013). Our machine learning model includes not only SNPs but also environmental and demographic variables so that we can identify how these factors jointly affect reading disability. Furthermore, we perform pathway and network analysis for the SNPs that are found by elastic net to investigate possible gene–gene interactions, as genes involved in the same biological process pathway will have functional interactions with each other and biological process pathways potentially interact.

Our study is performed by leveraging the large population‐based database, the Avon Longitudinal Study of Parents and Children (ALSPAC; Boyd et al., 2013), which is a longitudinal birth cohort from the UK. The ALSPAC is ideal for testing our initial hypotheses as it contains a large sample of children with genetic, environmental, demographic, and behavioral data. It is the largest publicly available genome‐wide data for reading disability. By applying the aforementioned proposed approach to ALSPAC data, our major findings include the association of novel genes and biological process pathways with reading disability. We also provide evidence that a combination of genetic, environment, and demographic factors was informative for predicting reading disability status, with some factors associated with having reading disability, while other factors were associated with not having reading disability. Lastly, we found that the biological process pathways interacted with each other, suggesting that the genetics of reading disability is a highly complex system.

## 2 MATERIALS AND METHODS

### 2.1 Participants

ALSPAC is a population‐based birth cohort which has been extensively described in various studies (Boyd et al., 2013; Eicher et al., 2013; Fraser et al., 2013; Paracchini et al., 2008). The total sample size was 15,454 pregnancies, resulting in 15,589 fetuses, and 14,901 were alive at 1 year of age. For this study, we used data from 8,071 participants who had behavioral data and genetic data. Measures included parent surveys and clinical data. Please note that the study website contains details of all the data that are available through a fully searchable data dictionary (http://www.bristol.ac.uk/alspac/researchers/our‐data/). Data from this study are available through ALSPAC upon approval by executive board.

The inclusion criteria were as follows: (a) no diagnosis of autism spectrum disorder, (b) normal hearing status at Focus at 7, (c) nonverbal intelligence >72 standard score on the Wechsler Intelligence Scales for Children (Wechsler, Golombok, & Rust, 1992), and (d) enough data to classify as case–control (i.e., child had a minimum of 80% of data necessary for classification). These criteria were based on prior studies that used the ALSPAC for genetic analysis (Eicher et al., 2014; Paracchini et al., 2008; Scerri et al., 2012). We used a more lenient nonverbal cutoff than past studies which used a cut‐off of 75 standard score. We used a cut‐off of 72 because this represents the common cut‐off of 75 minus the standard error measurement. Lastly, for twin pairs one child was randomly selected for analysis to achieve data independence, which resulted in 186 children being removed the analysis set. This was done for both monozygotic and dizygotic twin pairs.

### 2.2 Measures

#### 2.2.1 Demographics

Biological sex and birthweight in grams were reported at birth. Maternal education was obtained at 32 weeks' gestation and measures the highest degree the mother had obtained by that point: CSE (certificate of secondary education generally obtained by age 16)/none, vocational, O levels (ordinary‐level subject‐specific qualifications obtained at age 16), A levels (advanced‐level subject‐specific qualifications obtained by age 18, required for entry to college), and college degree (any degree beyond A levels). Child's ethnicity was reported by mothers at 32 weeks’ gestation and then ALSPAC classified responses as white or non‐white. Bilingual language status was obtained via parent report at Focus at 8 as monolingual or bilingual. Hearing functioning was measured via bone conduction at Focus at 7. Attention‐deficit/hyperactivity disorder (ADHD) status was determined at age 7 using parent and teacher questionnaires. ADHD status was coded by subtype inattentive, hyperactive‐impulsive, combined, or typical.

#### 2.2.2 Reading

Reading skill was measured during Focus at 7 and Focus at 9 using a combination of word reading, spelling, and connected text tasks. At Focus at 7 years, children completed the single word reading subtest on the Wechsler Objective Reading Dimensions (Rust, Golombok, & Trickey, 1993), an experimenter‐derived spelling task (Bryant, Nunes, & Barros, 2014), and a phoneme deletion task (Rosner & Simon, 1971). Nonword repetition was measured at Focus at 8 (Gathercole, Willis, Baddeley, & Emslie, 1994). At Focus at 9, children completed single word reading, nonword reading (Nunes, Byrant, & Olsson, 2003), and spelling tasks like the ones presented to those at Focus at 7 years but with new word/items. Additionally at age 9, children completed the Neale Analysis of Reading Ability (NARA) (Neale, McKAY, & Childs, 1986), which provided scores for reading rate, accuracy, and reading comprehension.

#### 2.2.3 Nonverbal intelligence

The WISC‐III (Focus at 8) yielded an estimate of nonverbal intelligence. Nonverbal intelligence was used to filter out children with potential cognitive impairments and was included in the sparse machine learning model to determine whether nonverbal intelligence was an important predictor of reading disability case–control status above and beyond genetics. Nonverbal IQ score was derived from the following subtests: Picture Completion, Coding, Picture Arrangement, Block Design, and Object Assembly.

#### 2.2.4 Language

Receptive language was assessed using the Wechsler Objective Language Dimensions Language Comprehension subtest (WOLD) (Rust, 1996). Vocabulary was measured using the WISC‐III vocabulary subtest (Wechsler et al., 1992). These language variables were used to compare the samples and included in sparse machine learning models to determine whether language ability was an important predictor of reading disability case–control status above and beyond genetics.

### 2.3 Classifying case–control status

We used single word reading at 7, spelling at 7, phoneme deletion, nonword repetition, single word reading at 9, nonword reading at 9, spelling at 9, NARA fluency, NARA accuracy, and NARA reading comprehension to classify children. We computed z‐scores within the whole dataset and then classified participants. Children were classified as cases if they scored less than −1 *z*‐score on three or more reading tasks. This classification was based on work by Eicher (Eicher et al., 2014; Paracchini et al., 2008; Scerri et al., 2012) using the ASLPAC dataset. We expanded the number of reading tasks considered so as to better represent the skills that reading disabilities affect. Using this classification, system yielded 1,215 cases and 6,586 controls. Table 1 provides descriptive statistics by group for demographic, reading, nonverbal intelligence, and language variables.

**TABLE 1:** Descriptive statistics for all included variables by group

Variable	Child's age at assessment	Dyslexia	Control
Sample size		1,215	6,856
Biological sex	Birth		
Male		714 (58.76)	3,400 (49.6)
Female		501 (41.2)	3,452 (50.4)
Missing^a^		<5	<5
Ethnicity	Birth		
White		1,081 (88.97)	6,013 (87.70)
Non‐White^a^		<5	19 (0.28)
Mother's highest education	32 weeks' gestation		
CSE		238 (19.59)	940 (13.71)
Vocational		137 (11.28)	535 (7.80)
O Levels		418 (34.40)	2,136 (31.16)
A Levels		228 (18.77)	1,532 (22.35)
Degree		75 (6.17)	1,005 (14.65)
Missing		119 (9.79)	708 (10.33)
Birthweight (in g)	Birth	3,434.30 (555.20) *n* = 1,136	3,438.01 (528.09) *n* = 6,492
Bilingual language	8		
Monolingual		962 (79.18)	4,234 (61.76)
Bilingual		16 (1.32)	70 (1.02)
Missing		237 (19.51)	2,552 (37.22)
ADHD status	7		
ADHD—Combined		18 (1.48)	23 (0.34)
ADHD—Inattentive		18 (1.48)	23 (0.34)
ADHD—Hyperactive‐impulsive		6 (0.49)	8 (0.12)
No ADHD		900 (74.07)	4,546 (66.31)
Missing		273 (22.47)	2,251(32.83)
Receptive Language	8	6.94 (1.75) *n* = 984	7.68 (1.93) *n* = 4,324
WISC Vocabulary	8	8.81 (3.41) *n* = 978	11.83 (4.28) *n* = 4,317
WISC Nonverbal IQ	8	94.56 (14.96) *n* = 974	102.55 (16.11) *n* = 2,544
Reading:			
Single word reading at 7	7	16.98 (5.41) *n* = 1,064	31.19 (7.67) *n* = 4,701
Phoneme deletion	7	10.87 (6.92) *n* = 1,063	22.67 (8.46) *n* = 6,704
Spelling at 7	7	12.31 (8.79) *n* = 1,000	29.48 (10.81) *n* = 4,647
Nonword repetition	8	5.56 (2.57) *n* = 982	7.74 (2.25) *n* = 4,320
Single word reading at 9	9	4.18 (2.26) *n* = 1,137	8.49 (1.42) *n* = 4,473
Nonword reading	9	2.47 (1.77) *n* = 1,131	5.99 (2.04) *n* = 4,473
Spelling at 9	9	5.73 (3.03) *n* = 1,128	11.53 (2.21) *n* = 4,472
NARA reading rate	9	92.71 (11.51) *n* = 1,057	109.03 (10.09) *n* = 4,014
NARA accuracy	9	86.89 (8.57) *n* = 1,064	108.95 (10.35) *n* = 4,018
NARA reading comprehension	9	86.96 (8.36) *n* = 1,064	104.34 (9.40) *n* = 4,018

Abbreviation: NARA, Neale Analysis of Reading Ability (Neale MD. *Neale Analysis of Reading Ability—Revised*. Windsor: NFER‐Nelson; 1997.)

^a^ The exact number for these cells cannot be provided because of the low count and these values may contain zero.

### 2.4 Genotyping

ALSPAC children were genotyped using the Illumina HumanHap550 quad chip genotyping platforms by 23andMe subcontracting the Wellcome Trust Sanger Institute, Cambridge, UK, and the Laboratory Corporation of America, Burlington, NC, US. The resulting raw genome‐wide data were subjected to standard quality control methods. Individuals were excluded on the basis of gender mismatches; minimal or excessive heterozygosity; disproportionate levels of individual missingness (>3%); and insufficient sample replication (IBD < 0.8). Population stratification was assessed by multidimensional scaling analysis and compared with HapMap II (release 22) European descent (CEU), Han Chinese, Japanese, and Yoruba reference populations; all individuals identified as non‐European ancestry were removed. SNPs with a minor allele frequency of <1%, a call rate of <95% or evidence for violations of Hardy–Weinberg equilibrium (*p* < 5E−7) were removed. Cryptic relatedness was measured as proportion of identity by descent (IBD > 0.1). Related subjects that passed all other quality control thresholds were retained during subsequent phasing and imputation. 9,115 subjects and 500,527 SNPs passed these quality control filters.

### 2.5 Statistical analysis

#### 2.5.1 SNP screening

Because we have a super‐high‐dimensional dataset in terms of the number of SNPs, a screening procedure was recommended, which warrants a more robust model than putting all the SNPs into a multivariate model to link with reading disability (Fan & Lv, 2008). Following this recommendation, we used genome‐wide association to screen SNPs. Genome‐wide association (GWA) was completed in PLINK (version v2.00a2LM) (Chang et al., 2015) and performed chromosome by chromosome. The top 100 SNPs genome‐wide before multiple testing correction and any surviving SNPs after multiple testing correction were included in the subsequent multivariate modeling. A similar screening method was used by other genetic studies employing lasso methods to identify informative SNPs (Cho, Kim, Oh, Kim, & Park, 2009).

In addition to SNPs, we also included, in the multivariate model, nonverbal IQ, vocabulary, receptive language score, ADHD status (inattentive only, hyperactive only, and combined type), birthweight, bilingual language status, mum's highest education, and child's ethnic background. This is to factor out the potential influence from these demographic, environmental, and behavioral variables on reading disability so that the direct association between SNPs and reading disability can be better revealed.

#### 2.5.2 Multivariate modeling by elastic net

We used an elastic net model to link reading disability (case vs. control) with the SNPs which survived the screening step, as well as demographic, environmental, and behavioral covariates. An elastic net is a regularized regression model to enable simultaneous variable selection (in our case, variables are the SNPs) in high‐dimensional setting (Fan & Lv, 2008; Zou, 2006). It adds two regularization terms to the loss function of an ordinary regression model: one L1‐norm regularization whose effect is to force the regression coefficients of small effects to be exactly zero, thus enabling variable/SNP selection; another L2‐norm regularization to make sure highly correlated SNPs are selected. There are two tuning parameters corresponding to the two regularization terms to balance with the loss function. Tuning parameters selection is typically done using cross‐validation (see below).

The ratio between case and control was high (1,215:6,856); therefore, we decided to use oversampling to guarantee a 1:1 ratio between groups when performing cross‐validation. We used fivefold cross‐validation to determine the best tuning parameters. In fivefold cross‐validation, the sample is split into five random groups, four of which are used to train the model and one for testing. This splitting repeats until every “fold” has served as the test set. Cross‐validation was performed 10 times to select tuning parameters. After the best tuning parameters were identified, the model was refit using all the data to generate coefficients.

#### 2.5.3 Pathway enrichment and network analysis

We mapped informative SNPs from the elastic net to genes using g:SNPense on g:Profiler (Ensembl 90, Ensembl Genomes 37, rev 1741 build date 2017–10–91) (Reimand et al., 2016). After mapping SNPs to genes, we performed enrichment analysis using g:GOSt on g:Profiler. g:Profiler was selected over similar tools because recent comparisons on the available tools showed that g:Profiler has the most up‐to‐date repository of pathways and draws from multiple curated sources (e.g., KEGG, Reactome). We selected the following settings on g:Profiler: Homo sapiens; significant only; size of functional category between 10 and 500; size of query 3; significance threshold—g:SCS threshold; gene ontology—biological process; and biological pathways—Reactome. However, enrichment analysis alone only provides what pathways are overrepresented in a gene list, it cannot tell us how these pathways interact. Therefore, we used Cytoscape to explore how the pathways were connected (Shannon et al., 2003). Cytoscape performs network analysis on biological pathways and produces visualizations and network statistics. We imported gmt files from g:Profiler into Cytoscape and used enrichment map with standard settings.

### 2.6 Ethical approval

Data came from the Avon Longitudinal Study of Parents and Children (ALSPAC). Ethical approval for the study was obtained from the ALSPAC Ethics and Law Committee and the Local Research Ethics Committees (Arizona State University Institutional Review Board). Consent and assent were obtained by ALSPAC staff at the time of data collection.

## 3 RESULTS

### 3.1 Multivariate modeling

No SNPs survived multiple test correction. Therefore, we selected 147 SNPs which represented the top 100 SNPs genome‐wide before multiple test correction and 47 SNPs associated with reading disability from prior studies. We removed three SNPs for our list because one of the top 100 SNPs was an imputated marker and two SNPs from the previous literature were not genotyped within the ALSPAC sample (rs93434 and rs454942). The final list contained 145 SNPs (see Appendix for list of SNPs included in the model). We applied the elastic net model to the SNPs without and with demographic, environmental, and behavioral variables. We call these “Analysis_1” and “Analysis_2,” respectively, hereafter. Ninety‐one and 68 SNPs were identified with main effects in Analysis_1 and Analysis_2, respectively. Table 2 presents the results of the elastic net. Across the two analyses, there were 65 SNPs commonly selected. For Analysis_1, 57 SNPs were positively associated with reading disability case status (i.e., risk SNPs) and 34 SNPs were negatively associated with reading disability case status (i.e., protective SNPs). For Analysis_2, 46 SNPs were positively associated with reading disability, 22 were negatively associated, and seven demographic, environmental, and behavioral variables were selected. SNPs were selected from across the genome with the majority on chromosome 6.

**TABLE 2:** Reading disability‐associated SNPs identified with the ten largest main effects by elastic net model

SNP	Chr	Gene(s)	Coefficient	Variant type
Analysis 1				
rs3095073	4	MSANTD1	−0.4267	NMD transcript, intron
rs807701	6	DCDC2	0.0882	Intron
rs56364346	6	ZNF165	0.0073	Intron
rs9368549	6	ZNF165	0.0260	Intron
rs9393886	6	ZNF165	0.0629	Intron
rs7765678	6	DCDC2	−0.0275	Intron
rs4504469	6	KIAA0319	−0.0499	Missense
rs2038137	6	KIAA0319	−0.0278	5′ UTR, intron
rs35491132	6	.	0.0062	
rs17750424	6	.	−0.2481	
rs1225598	6	.	−0.1412	
rs149990	6	.	−0.0786	
rs13193542	6	.	−0.0374	
rs34064842	6	.	−0.0332	
rs13212318	6	.	−0.0080	
rs2710102	7	CNTNAP2	0.0483	Intron, noncoding exon
rs2268119	12	GRIN2B	0.0143	Intron
rs2192973	12	GRIN2B	−0.3909	Intron
rs1012586	12	GRIN2B	−0.2089	Intron
rs78361609	13	USP12	0.7748	Intron
rs10046	15	CYP19A1,MIR4713HG	0.9929	3′ UTR, intron, noncoding exon
rs1075938	15	DNAAF4	0.1034	5′ UTR
rs1065778	15	CYP19A1,MIR4713HG	−0.1647	Intron, noncoding transcript
rs12606138	18	NEDD4L	0.1881	Intron, noncoding exon
rs2516536	22	THAP7‐AS1,AC002472.2	0.0170	Intron, noncoding transcript
rs5965871	X	.	0.0545	
Analysis 2				
rs17763089	6	HIST1H1B	0.0206	
rs2143340	6	TDP2	0.0278	
rs13199906	6	.	0.2154	

Shared					
			Analysis 1	Analysis 2	
rs114425071	1	SLC25A33	1.1411	1.1439	Intron
rs72946339	1		1.2778	1.0341	
rs34170608	2	EML6	0.1831	0.0831	Intron, noncoding exon
rs2114648	2	SSB	0.3723	0.5152	Intron
rs76229518	2	AC064875.1	−0.5668	−0.3955	Intron, noncoding transcript
rs1401776	2	.	−0.3046	−0.2950	
rs16866459	2	.	0.4207	0.4156	
rs6772326	3	LINC01208	1.6061	2.0254	Intron, noncoding exon
rs2119748	3	ROBO2	0.2119	0.2497	NMD transcript, intron
rs362279	4	MSANTD1	0.2208	−0.2162	NMD transcript, intron
rs7685028	4	RAPGEF2	−0.1650	−0.0369	Intron
rs6828649	4	RAPGEF2	−0.0219	−0.0164	Intron
rs7692595	4	RAPGEF2	−0.0186	−0.1432	Intron
rs142829912	4		1.0840	1.2860	
rs112071915	5	AC113414.1	0.5224	0.4631	Intron, noncoding exon
rs143602479	5	CWC27	0.6629	0.7166	Intron, noncoding exon
rs6871223	5	.	−0.3706	−0.4572	
rs807724	6	DCDC2	0.0479	−0.1672	Intron
rs6935076	6	KIAA0319	0.0359	−0.0384	Intron
rs16889506	6	KIAA0319	0.0645	0.0323	Intron
rs9393885	6	ZNF165	0.0926	0.0163	Intron
rs2274305	6	DCDC2	−0.1273	0.0645	Missense, intron
rs3765502	6	DCDC2	−0.1156	−0.1422	Intron
rs793862	6	DCDC2	−0.0305	0.0254	Intron, NMD transcript
rs16889556	6	KIAA0319	−0.1231	−0.0335	Intron
rs699463	6	KIAA0319	−0.0589	−0.0889	3′ UTR
rs200257294	6	.	0.0700	0.0538	
rs201193697	6	.	0.3387	0.1779	
rs149150340	6	.	0.4891	0.0058	
rs7782412	7	FOXP2	0.0034	0.0638	NMD transcript, intron, noncoding exon
rs936146	7	FOXP2	0.0696	0.0997	NMD transcript, intron
rs147278887	7	RELN	5.6215	1.8715	Intron
rs188260392	7	ZNF804B	0.7608	0.5216	Intron
rs923875	7	FOXP2	−0.0394	0.0292	5′ UTR, NMD transcript, noncoding transcript
rs113178744	7	.	0.5440	0.6075	
rs112276179	8	DLC1	0.2419	0.1987	NMD transcript, noncoding transcript
rs148138267	8	.	1.0566	1.2448	
rs7093764	10	MALRD1	0.2547	0.1670	NMD transcript
rs1163203	10		0.0754	0.1718	
rs1079727	11	DRD2	0.1250	0.0233	NMD transcript, noncoding transcript
rs145953567	11	IGHMBP2	0.8748	0.8987	NMD transcript, noncoding transcript
rs9634041	11	.	0.2866	0.2924	
rs5796555	12	GRIN2B	0.1810	0.0754	NMD transcript
rs2216128	12	GRIN2B	0.5199	0.0248	NMD transcript
rs116902441	12	SPPL3	0.5781	0.7470	NMD transcript
rs115332388	12	.	0.2115	0.2601	
rs146055250	12	.	1.6433	2.2141	
rs77527164	13	USP12	0.0540	0.2212	NMD transcript
rs116921729	13	USP12	−0.4395	0.0902	NMD transcript
rs180701414	14	LINC02291	1.4980	1.2601	NMD transcript, noncoding transcript
rs57809907	15	DNAAF4,DNAAF4‐CCPG1	0.2301	0.0554	3′ UTR, 5′ UTR, NMD transcript, noncoding transcript
rs2289105	15	CYP19A1,MIR4713HG	−0.6717	−0.0591	Intron, noncoding transcript
rs2899472	15	CYP19A1,MIR4713HG	−0.1124	−0.1302	Intron, noncoding transcript
rs1902586	15	CYP19A1,MIR4713HG	−0.1073	−0.1877	Intron, noncoding transcript
rs77641439	15	DNAAF4,DNAAF4‐CCPG1	−0.2433	−0.1850	3′ UTR, NMD transcript, intron, missense, noncoding transcript
rs3743205	15	DNAAF4,DNAAF4‐CCPG1	−0.1990	−0.1400	5′ UTR, NMD transcript, noncoding exon
rs12899331	15	.	0.0177	−0.0157	
rs11860694	16	ATP2C2	0.0414	0.1119	Intron, noncoding exon
rs6564903	16	CMIP,AC092135.1	0.0046	0.0469	Intron, missense, noncoding exon
rs11873029	18	DYM	0.0229	−0.0581	Intron
rs8094327	18	NEDD4L	−0.1725	−0.0966	Intron, noncoding exon
rs1299348	18	.	0.0208	0.0804	
rs182460592	19	CLEC17A	1.4398	1.3699	NMD transcript, intron
rs459962	21	SAMSN1,SAMSN1‐AS1	−0.0229	−0.0078	Intron, noncoding transcript
rs112331442	22	THAP7‐AS1,AC002472.2	−0.1769	−0.1051	Intron, noncoding transcript

Abbreviation: Chr, chromosome.

Compared to a null model, the model in Analysis_1 fits the data significantly better (*F* (1, 80) = 6.49, *p* < .001). The risk SNPs were located within 29 genes with the majority representing intron variants or noncoding variants; however, several SNPs were reported to have more than one variant effect. For example, rs57809907 mapped to *DNAA4F* (ENSG00000256061; chr15:55430684) with variant effects in the 3′ UTR and nonsense‐mediated decay variant effect. The protective SNPs were located within 16 genes with the majority of variant effects being intronic or noncoding. There were eight genes present in both the risk and protective gene lists. Additionally, many of the SNPs were located within the same gene. For example, there were four intron variant SNPs located within *ZNF165* (chr 6).

Compared to a null model, the model in Analysis_2 fits the data significantly better (*F* (1, 76) = 10.35, *p* < .0001). The risk SNPs were located within 23 genes, while the protective SNPs were located within 13 genes. Non‐white children via parent‐reported ethnicity, monolingual language status, better receptive language skills, higher nonverbal IQ, increased mother's highest education, and better vocabulary skills were all associated with typical reader status, while low birthweight was associated with reading disability status. Examination of sample distributions suggests that ethnicity and bilingual language status were selected due to higher number for monolingual and non‐white children in the typical reader group; therefore, these features may not be as informative as the language features. Although all children of non‐European ancestry were removed for the analyses, a small minority of parents still reported their children as “non‐white.”

We compared the results from both analyses to identify which SNPs replicated internally and map the replicated SNPs to genes. The 60 SNPs common to both Analysis_1 and Analysis_2 mapped to 31 genes, including the candidate genes *FOXP2* (chr 7) and *DCDC2* (chr 6).

### 3.2 Pathway analysis

For Analysis_1, the risk gene list was significantly overrepresented in fourteen biological process pathways after multiple testing correction (false discovery rate), while the protective gene list was significantly overrepresented in three biological process pathways. There was one pathway shared by both lists, neuron migration. For Analysis_2, the risk gene list was overrepresented in seven biological process, while the protective gene list was overrepresented in three biological process pathways. All overrepresented pathways were associated with brain and/or dendrite development. Table 3 presents information for all pathways identified.

**TABLE 3:** Overrepresented pathways from g:Profiler

GO term	Pathway function	*q*‐value	Associated with
Analysis_1			
GO:0030900	Forebrain development	0.0000	RD
GO:0001764	Neuron migration	0.0000	RD
		0.0000	TR
GO:0021537	Telencephalon development	0.0000	RD
GO:0120035	Regulation of plasma membrane‐bounded cell projection organization	0.0002	TR
GO:0031344	Regulation of cell projection organization	0.0002	TR
GO:0007420	Brain development	0.0004	RD
GO:0060322	Head development	0.0006	RD
GO:0120035	Regulation of plasma membrane‐bounded cell projection organization	0.0016	RD
GO:0031344	Regulation of cell projection organization	0.0018	RD
GO:0007417	Central nervous system development	0.0045	RD
GO:0021987	Cerebral cortex development	0.0066	RD
GO:0031345	Negative regulation of cell projection organization	0.0074	TR
GO:0021543	Pallium development	0.0134	RD
GO:0050808	Synapse organization	0.0236	RD
GO:0050767	Regulation of neurogenesis	0.0343	RD
GO:0007611	Learning or memory	0.0385	RD
GO:0048812	Neuron projection morphogenesis	0.0442	RD
Analysis_2			
GO:0001764	Neuron migration	0.0000	TR
		0.0000	RD
GO:0030900	Forebrain development	0.0003	RD
GO:0007420	Brain development	0.0012	RD
GO:0060322	Head development	0.0017	RD
GO:0021537	Telencephalon development	0.0026	RD
GO:0120035	Regulation of plasma membrane‐bounded cell projection organization	0.0036	TR
GO:0031344	Regulation of cell projection organization	0.0038	TR
GO:0007417	Central nervous system development	0.0128	RD
GO:0050808	Synapse organization	0.0467	RD

Abbreviations: RD, reading disability; TR, typical reading.

### 3.3 Network analysis

**FIGURE 1:**
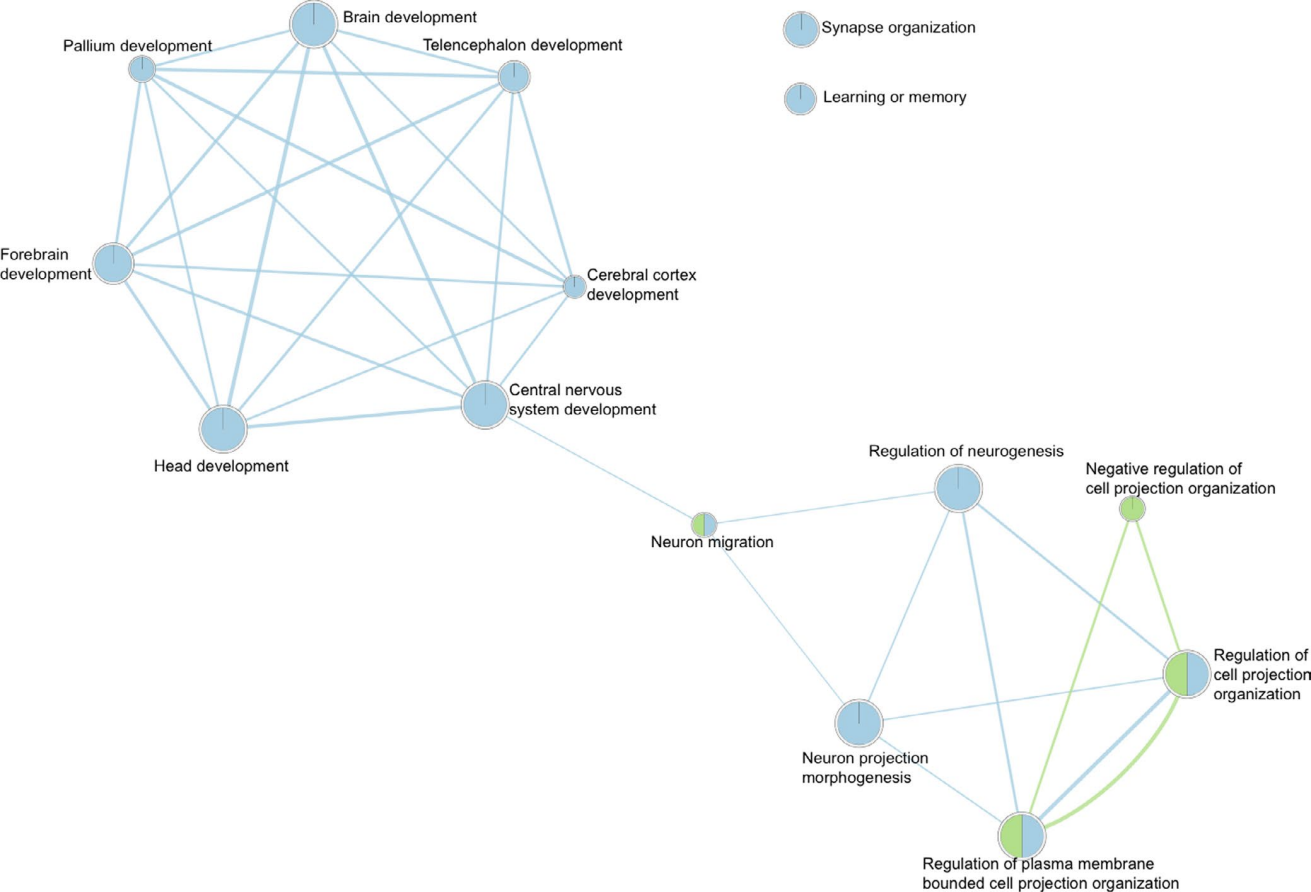
This figure is a graphical display of the relationships between overrepresented biological process using gene lists from Analysis_1. Biological process pathways are represented by circles, called nodes. Relationships between pathways are presented by lines, called edges. The more statistically powerful the edge between two nodes, the wider the edge. Blue nodes are biological pathways which were overrepresented for on the dyslexia risk gene list, while green nodes were overrepresented on the protective gene list. Nodes significantly overrepresented in both gene lists are therefore colored blue and green

**FIGURE 2:**
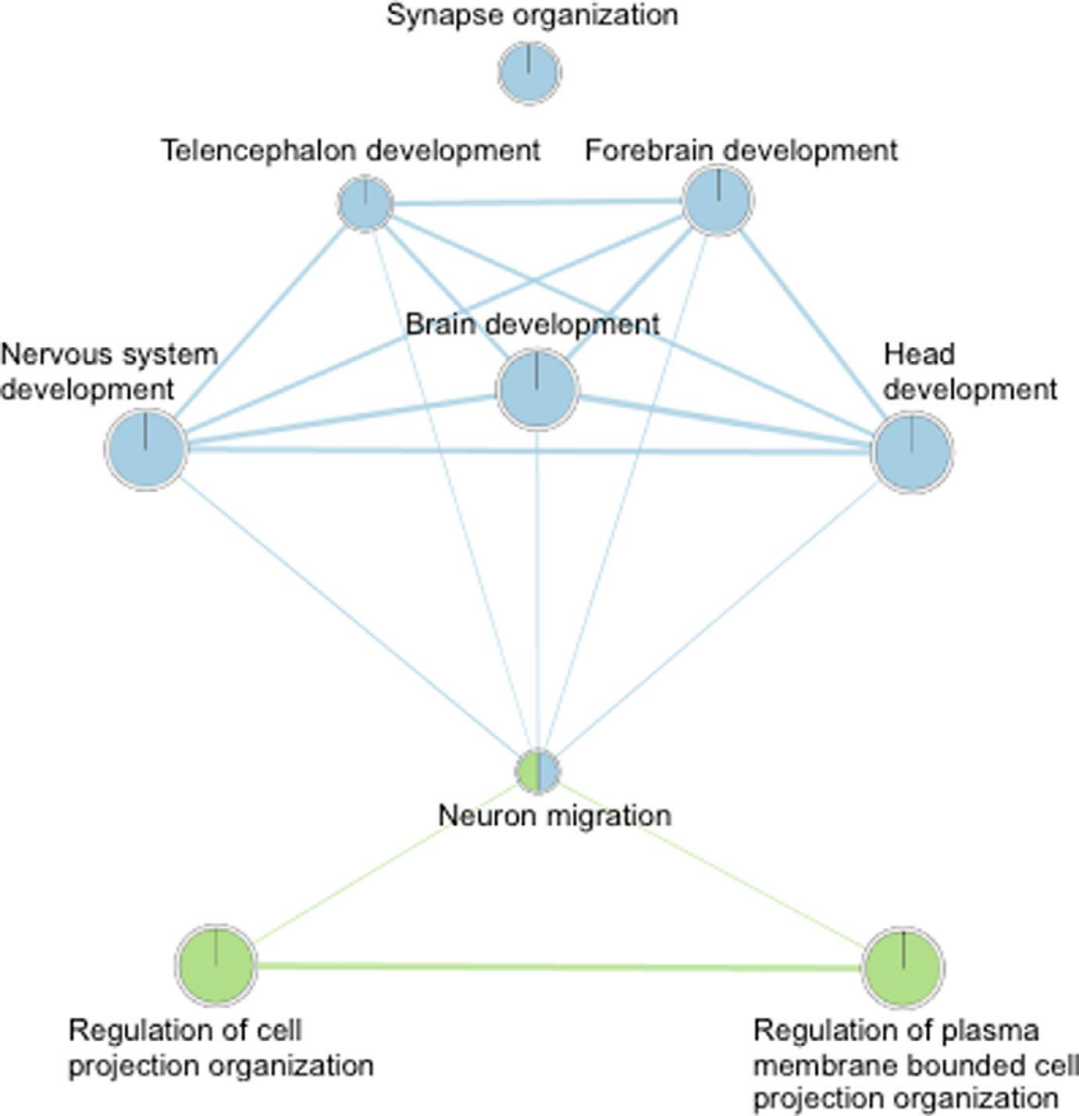
This figure is a graphical display of the relationships between overrepresented biological process using gene lists from Analysis_2. Biological process pathways are represented by circles, called nodes. Relationships between pathways are presented by lines, called edges. The more statistically powerful the edge between two nodes, the wider the edge. Blue nodes are biological pathways which were overrepresented for on the dyslexia risk gene list, while green nodes were overrepresented on the protective gene list. Nodes significantly overrepresented in both gene lists are therefore colored blue and green

We imported the pathway results from g:Profiler into Cytoscape for Analysis_1 and Analysis_2, and performed network analysis. Figures 1 and 2 visualize the relationships between the pathways with only highly relevant connections between pathways shown (i.e., edges with a weight >0.4). The network analysis results indicated that there were relevant edges between most of the pathways with neuron migration serving as a central node between two clusters of pathways. The pathways in blue in Figures 1 and 2 were overrepresented by genes positively associated with reading disability, while the pathways in green were overrepresented by genes negatively associated with reading disability. The network analysis suggests that the biological pathways associated with reading disability case–control status are linked together.

## 4 DISCUSSION

We investigated the genetic and environmental contributions to reading disability using a combination of GWAS, sparse machine learning, and pathway and network analysis. Using the pipeline in this study, we were able to overcome a common limitation of genetic studies of reading disability, namely lack of significant findings after multiple testing correction. There are three major findings from this study. We identified novel genes associated with reading disability case–control status, some of which were indicative of risk and others were protective. We provide evidence that a combination of genetic, environment, and demographic factors is informative for predicting reading disability case–control status. Lastly, we found that biological process pathways associated with reading disability case–control status interacted with each other, suggesting that the genetics of reading disability is a highly complex system.

### 4.1 Novel genes

The elastic net models selected SNPs genome‐wide as informative for predicting reading disability case–control status. We compared results between the two models in Analysis_1 and Analysis_2 and found that there were 60 SNPs shared by both model results, 41 positively associated with reading disability and 19 negatively associated with reading disability. We found that there were SNPs associated with reading disability and typical reader status. Multiple SNPs from *KIAA0319* (Zhao, Chen, Zhang, & Zuo, 2016), *DCDC2* (Meng et al., 2005), and other previously identified genes were selected by the elastic net model, providing replication and further support for these genes being involved in reading disability (Poelmans, Buitelaar, Pauls, & Franke, 2011).

We associated 18 novel genes with reading disability case–control status. Of these, *RAPGEF2*, *DLC1*, *TDP1*, and *RELN* were highly represented in the enriched biological process pathways and are therefore more likely to be involved in the biology of reading disability. The combination of the elastic net findings and the pathway analyses indicate that SNPs in these four genes play a larger role in reading disability case–control status that previously assumed. *RAPGEF2* (chr4q32.1) is a guanine nucleotide exchange factor and is involved in neuron migration and brain development (Maeta et al., 2018). *RAPGEF2* helps with the formation of the major forebrain fiber connections for the corpus callosum, the anterior commissure, and the hippocampal commissure during brain development. *RAPGEF2* has been associated with decrease in the ability to learn and deficits in working memory in knockout mouse models (Maeta et al., 2018). This is the first study to associate markers from *RAPGEF2* with reading disability in humans. *DLC1* (chr 8p22) encodes a GTPase‐activating protein that terminates downstream signaling of GTPases RHOA, RHOB, RHOC, and CDC42 and plays a critical role in cell migration and proliferation. Activation of *DLC1* increases cell migration, but reduces directionality (Tai et al., 2008). *DLC1* has a CpG island making it a target for methylation and gene silencing. *DLC1* has most frequently been linking to colon cancer, but work in mouse models has shown that it is a critical gene for brain development during embryogenesis (Durkin et al., 2005). *TDP2* (chr6p22.3) is involved in DNA repair and protects transcription of genes necessary for neurological development (Hornyak et al., 2016). *TDP2* is also known as *TTRAP* and is located within the DYX2 loci and near *DCDC2*, a frequently reported gene associated with reading disability (Poelmans et al., 2011). Prior work on *DCDC2* has associated SNPs from *TDP1*/*TTRAP* with reading disability (Cope et al., 2012; Deffenbacher et al., 2004; Meng et al., 2005). Given their proximity, it is likely that *TDP1* and *DCDC2* interact with each other. *RELN* (chr7q22.1) is a protein encoding gene which provides instruction for reelin (Devasenapathy et al., 2018). Reelin is expressed in the brain before and after birth and activates the reelin signaling pathway (Folsom & Fatemi, 2013). The reelin signaling pathway is responsible for neuron migration, as well as axon maintenance and neuronal signaling in adulthood (Folsom & Fatemi, 2013). One previous study has associated a triallelic unit in the *RELN* gene to a family with reading disability and the interactions between *RELN*, *DCDC2*, and *ROBO1* (Devasenapathy et al., 2018). Additionally, change in *RELN* which decrease the production of reelin has been associated with autism spectrum disorder and other neurodevelopmental disorders (Folsom & Fatemi, 2013; Ishii, Kubo, & Nakajima, 2016). As we excluded all children with suspected autism spectrum disorder, we can infer that changes in *RELN* have broader effects than just deficits in social interaction and communication, but may also affect general learning structures and functions. *RAPGEF2*, *TDP1*, *DLC1*, and *RELN* all have roles in brain development, function, and maintenance which align with genes more frequently associated with reading disability (e.g., *KIAA0319*, *DCDC2*). These findings provide some insight into the underlying biology of reading disability.

Several genes had multiple SNPs selected by the elastic net; however, SNPs from the same gene did not always have the same directionality. For example, *KIAA0319* had six SNPs selected as informative. Of these, two were found to be positively associated with reading disability (rs6935076 and rs16889506) and four were found to be negatively associated with reading disability (rs16889556, rs699463, rs4504469, and rs2038137). One interpretation of this is that some SNPs increase risk of developing reading disability, while other offer protection from reading disability. This interpretation would fit in with recent findings that there are protective alleles for reading disability (Powers et al., 2016; Shao, Niu, et al., 2016). An alternative solution is that dichotomizing reading ability may serve as a barrier to understanding the genetics of reading (dis)ability. This is an avenue for future research.

### 4.2 Environment and demographic factors

Alongside SNPs, there were environmental and demographic features that were informative for reading disability case–control status. Of these, better receptive language skills, higher nonverbal IQ, increased mother's highest education, and better vocabulary skills were all associated with typical reader status, while low birthweight was associated with reading disability status. All of these factors have been previously linked to reading disability and reading ability. Receptive language skills and vocabulary are both positively predictive of reading comprehension (Berninger, Abbott, Vermeulen, & Fulton, 2006; Braze et al., 2016; Language & Reading Research Consortium, 2015; Tunmer & Chapman, 2012). Other research has demonstrated early receptive language and vocabulary abilities are predictive of reading disability status (Lyytinen et al., 2004; Stojanovik & Riddell, 2008; Torppa et al., 2010; Van Der Leij et al., 2013). Research exploring children at risk of reading and academic difficulties has shown that maternal education works as a preventative measure for difficulties (Dollaghan et al., 1999; Lervåg, Dolean, Tincas, & Melby‐Lervåg, 2019), but this relationship is complicated (Harding, Morris, & Hughes, 2015). Additionally, for children at family risk of reading disability nonverbal IQ can serve as a protective factor. Lastly, lower birthweight has been associated with a higher risk of reading disability (Mascheretti et al., 2013, 2018), although the exact nature of this association requires more exploration. Considering the number of genes and biological processes that are linked to reading disability that play a role in brain development before and after birth, any factors which could impair brain development are likely to increase risk for reading disability. In summary, our findings regarding the influence of selected environmental and demographic factors support past research and the assumption that genes potentially with environment and demographic factors.

### 4.3 Biological process

Our analyses identified several significantly enriched biological processes, including neuron migration, nervous system development, and dendrite development and regulation. Some of these pathways, like neuron migration, have been previously linked to reading disability (Carrion‐Castillo, Franke, & Fisher, 2013; Poelmans et al., 2011), while others have not. Additionally using network analysis, we mapped the interactions between pathways. The network analysis revealed that all of the identified pathways interacted with each other either directly or mediated by neuron migration or nervous system development.

Neuron migration (GO:0001764) was the most overrepresented biological process pathway and was identified for both the risk and protective gene lists. Neuron migration is a large biological process pathway that is responsible for organizing neuronal structure and organization in the developing brain. This pathway encompasses a number of smaller pathways, including the reelin signaling pathway discussed earlier. Neuron migration has been previously associated with reading disability in multiple samples (Luciano, Gow, Pattie, Bates, & Deary, 2018; Poelmans et al., 2011); therefore, we provide replication of this finding. Neuronal migration has been hypothesized to be an underlying biological etiology of reading disability because of the differences in brain structure and function in people with reading disability compared to typical readers (McCandliss & Noble, 2003; Niogi & McCandliss, 2006; Waldie et al., 2017). Our evidence for neuron migration fits in with current research examining the gene–brain–behavior model (Landi & Perdue, 2019). An extension from our study is that it is possible that neuron migration was shared for positive and negative associated genes suggesting that the genetic architecture of reading is shared for reading disability and typical reading which raises new questions about the development of reading.

Beyond neuron migration, we also identified several pathways associated with brain development and regulation of dendrite development. The brain development pathways were enriched in the risk gene sets and whose primary roles are structural development from formation to mature structure (e.g., forebrain development, telencephalon development). The dendrite regulation pathways were enriched in protective gene set. The dendrite regulation pathways were primarily responsible for creating axons (e.g., regulation of plasma membrane bounded cell projection organization) and neurons in early development and then maintaining axon and neuron functions throughout the lifespan. Both sets of pathways, brain development and dendrite regulation, interacted with neuron migration furthering the possibility that there is a shared genetic and neurobiological architecture for reading. Additionally, the fact that all of the pathways are associated with brain development furthers the gene–brain–behavior model under investigation (Landi & Perdue, 2019).

### 4.4 Limitations and future directions

There are a number of limitations for this study. First, several of SNPs selected by the elastic net model were imputed during genotyping. This suggests that either our screening methods were not stringent enough or the model had a tendency to select markers which were correlated due to location. This was overcome by using pathway analysis in conjunction with elastic net as imputed genes were dropped from pathway analysis. Second, reading is not a dichotomous skill—our phenotype does not reflect the nature of reading and dichotomizes a continuous skill using an arbitrary cut point. This decision results in two problems: (a) We are not adequately reflecting the nature of reading and (b) we decrease power by creating a smaller group of cases. Although our case–control design matches prior research, we recognize these limitations. We attempted to overcome these limitations by using oversampling methods to compensate for the unbalanced design. Third, our model could not quantify interactions between genes and environmental/demographic factors. Instead, our model examined the combined influence, but not how they interacted. This is a limitation that can be overcome in future analyses using alternative models and/or databases. Fourth, this is a small sample size in terms of genetic studies overall, but this sample size is comparable to genetic studies for dyslexia or reading disability. The goal of this study was to test our pipeline and determine some pilot results using a well‐known database. Lastly, our findings, especially the novel genes and biological pathways, need to be replicated in other studies with more diverse genetic populations. The ALSPAC database is a great database for testing new methods and obtaining discovery findings because of its size, breadth of measures, and high data quality, but findings from it can only be generalized to Caucasian genetic and English‐speaking populations. Reading, however, is a worldwide skill and to truly understand the genetic, environmental, and demographic factors of reading (dis)ability we need to include samples with diverse languages, socioeconomic backgrounds, and genetic ethnicity. Future studies should examine the genetics of reading ability, in addition to the genetics of reading disability versus typical reading. This approach would be similar to how researchers are examining educational attainment and general cognitive ability (Rabinowitz et al., 2019).

### 4.5 Conclusion

Our study suggests there are multiple genes associated with reading disability case–control status, which aligns with numerous studies (Eicher et al., 2014; Fisher et al., 1999; Gu et al., 2018; Hofmeister et al., 2015; Poelmans et al., 2011; Scerri et al., 2011). Our analysis approach allowed us to investigate the impact of multiple genetic markers without losing data due to multiple test correction. In doing so, we identified several novel genes. We provided evidence that mother's education and child language skills may provide protection from genetic risk. Additionally, our pathway and network analyses indicated that neuron migration, brain development pathways, and dendrite regulation pathways are associated with reading disability case–control status and that brain development and dendrite regulation pathways interact with each other through neuron migration. Our results support the hypothesis that reading disability represents a complex system with multiple genes, environmental, and demographic factors involved in an interactive fashion. Furthermore, our results suggest that there is a shared genetic and neurobiological architecture for reading (dis)ability which requires more research.

## CONFLICT OF INTEREST

The authors declare that they had no financial or commercial conflicts of interest.

## AUTHOR CONTRIBUTION

HSL conceived and designed the study under the mentorship of JL and VD. HSL analyzed and interpreted the data. XL provided critical support for analyzing the data. HSL drafted the manuscript with input and suggestions from JL and VD. HSL approved the final version of the manuscript on behalf of all the authors.

### Peer Review

The peer review history for this article is available at https://publons.com/publon/10.1002/brb3.1735.

## Data Availability

Please note that the study website contains details of all the data that are available through a fully searchable data dictionary (http://www.bristol.ac.uk/alspac/researchers/our‐data/). Data from this study are available through ALSPAC upon approval by executive board.

## APPENDIX 1

Genomic position and association testing results for SNPs included in elastic net

**Table:**

rs ID number	Chr	Position	Odds risk	*p* (unadjusted)
rs114425071	1	9,608,828	2.9504	.0001
rs72946339	1	84,897,639	4.5326	.0001
rs16866459	2	7,953,279	1.5329	.0001
rs76229518	2	13,109,516	0.5312	.0001
rs1401776	2	36,099,141	0.7412	.0001
rs34170608	2	55,034,082	1.2269	.0001
rs2114648	2	170,654,352	0.7657	.0001
rs2119748	3	77,255,415	1.2510	.0001
rs6772326	3	176,409,280	5.5539	.0001
rs362279	4	3,260,072	0.8241	.0001
rs4690081	4	3,262,105	0.8246	.0001
rs3135169	4	3,262,374	0.8247	.0001
rs3095073	4	3,263,138	0.8249	.0001
rs142829912	4	12,136,509	3.7553	.0001
rs184866744	4	96,367,853	2.43E+19	.0001
rs7692595	4	160,128,440	0.8285	.0001
rs6828649	4	160,128,857	0.8286	.0001
rs7685028	4	160,132,990	0.8284	.0001
rs143602479	5	64,276,829	2.3051	.0001
rs6871223	5	134,821,336	0.7097	.0001
rs112071915	5	162,000,806	1.5959	.0001
rs35491132	6	27,527,227	1.2945	.0001
rs34064842	6	27,688,625	1.2952	.0001
rs13212318	6	27,688,841	1.2951	.0001
rs13202295	6	27,698,837	1.2951	.0001
rs13202291	6	27,698,857	1.2951	.0001
rs17750424	6	27,701,122	1.2949	.0001
rs13193542	6	27,702,425	1.2948	.0001
rs13199906	6	27,834,139	1.2945	.0001
rs17763089	6	27,835,218	1.2945	.0001
rs17695758	6	27,837,183	1.2945	.0001
rs149583087	6	27,912,437	1.3117	.0001
rs142965311	6	27,989,252	1.2999	.0001
rs138234416	6	27,992,898	1.2999	.0001
rs71559067	6	27,994,416	1.2999	.0001
rs149990	6	27,998,258	1.2722	.0001
rs13193295	6	28,003,228	1.2998	.0001
rs149150340	6	28,012,278	1.2998	.0001
rs201193697	6	28,018,686	1.6011	.0001
rs9393885	6	28,050,009	1.2283	.0001
rs9393886	6	28,050,039	1.2283	.0001
rs9368549	6	28,050,047	1.2283	.0001
rs56364346	6	28,050,762	1.2283	.0001
rs9357061	6	28,051,772	1.2283	.0001
rs9368550	6	28,051,803	1.2283	.0001
rs2295594	6	28,053,097	1.2283	.0001
rs9357062	6	28,054,404	1.2283	.0001
rs16893666	6	28,054,707	1.2283	.0001
rs2273564	6	28,057,594	1.2283	.0001
rs1853097	6	28,058,635	1.2283	.0001
rs9393888	6	28,059,217	1.2283	.0001
rs3734573	6	28,059,437	1.2283	.0001
rs9357063	6	28,060,005	1.2283	.0001
rs3823180	6	28,061,744	1.2283	.0001
rs9368551	6	28,061,792	1.2283	.0001
rs34152964	6	28,062,442	1.2283	.0001
rs57311580	6	28,062,639	1.2283	.0001
rs9393890	6	28,063,855	1.2283	.0001
rs9380052	6	28,064,623	1.2283	.0001
rs9366715	6	28,064,633	1.2283	.0001
rs9380054	6	28,067,537	1.2283	.0001
rs2116981	6	28,067,951	1.2283	.0001
rs9368552	6	28,068,426	1.2283	.0001
rs145806375	6	28,069,621	1.2284	.0001
rs2281588	6	28,072,602	1.2283	.0001
rs200257294	6	28,074,526	1.3649	.0001
rs34131763	6	28,075,000	1.2283	.0001
rs17711344	6	28,077,602	1.2284	.0001
rs36078605	6	28,078,032	1.2283	.0001
rs6931858	6	28,078,411	1.2283	.0001
rs9393891	6	28,079,160	1.2283	.0001
rs9468286	6	28,079,428	1.2283	.0001
rs9393892	6	28,081,394	1.2283	.0001
rs9380055	6	28,081,629	1.2283	.0001
rs9368553	6	28,082,265	1.2283	.0001
rs9368554	6	28,082,711	1.2283	.0001
rs4713137	6	28,083,521	1.2283	.0001
rs9348793	6	28,084,189	1.2283	.0001
rs1225598	6	28,160,799	1.2271	.0001
rs188260392	7	88,921,196	2.5722	.0001
rs147278887	7	103,229,564	24.8636	.0001
rs113178744	7	125,404,729	1.7758	.0001
rs142918851	7	138,322,989	855.7270	.0001
rs112276179	8	13,033,831	1.3125	.0001
rs148138267	8	15,744,831	3.0408	.0001
rs7093764	10	19,445,888	1.2054	.0001
rs145953567	11	68,704,832	2.4295	.0001
rs9634041	11	69,684,178	1.2923	.0001
rs115332388	12	13,006,964	1.2365	.0001
rs148819926	12	79,898,068	8.37E+06	.0001
rs146055250	12	104,754,884	5.4094	.0001
rs116902441	12	121,255,085	1.6642	.0001
rs78361609	13	27,681,745	1.5264	.0001
rs116921729	13	27,691,016	1.5272	.0001
rs77527164	13	27,699,000	1.4666	.0001
rs180701414	14	98,118,338	6.7931	.0001
rs182460592	19	14,703,621	6.4912	.0001
rs112331442	22	21,358,723	0.8286	.0001
rs2516536	22	21,362,474	1.2003	.0001
SNPs from previous literature				
rs793862	6	24,207,200	1.0376	.4876
rs807701	6	24,273,791	1.0646	.2028
rs807724	6	24,278,869	1.0540	.3500
rs2274305	6	24,291,203	1.0706	.1680
rs7765678	6	24,330,544	1.0134	.8818
rs3765502	6	24,354,045	0.9381	.3994
rs699463	6	24,544,903	0.9655	.4768
rs16889506	6	24,595,853	1.1278	.0364
rs16889556	6	24,641,605	0.8569	.0151
rs6935076	6	24,644,322	0.9655	.4768
rs2038137	6	24,645,943	0.9967	.9462
rs2143340	6	24,659,071	1.0119	.8591
rs4504469	6	24,588,884	0.9551	.3409
rs923875	7	113,735,036	0.9614	.4174
rs7782412	7	114,290,415	0.9976	.9634
rs2710102	7	147,574,390	0.9701	.5208
rs936146	7	114,294,405	1.1149	.0247
rs1163203	10	70,554,635	1.0836	.2402
rs1079727	11	113,289,182	1.0693	.2990
rs5796555	12	13,855,534	0.9658	.5321
rs1012586	12	13,855,632	0.9442	.2965
rs2268119	12	13,872,634	0.9127	.1517
rs2216128	12	13,883,014	0.9066	.0927
rs2192973	12	13,896,555	0.9109	.1133
rs2289105	15	51,507,508	1.0352	.4627
rs8034835	15	51,512,664	1.0395	.4118
rs2899472	15	51,516,055	0.9270	.1621
rs1902586	15	51,570,853	0.9517	.6768
rs77641439	15	55,722,872	0.7897	.0053
rs57809907	15	55,722,882	1.1179	.1683
rs3743205	15	55,790,530	1.0442	.6682
rs1075938	15	55,790,691	1.2646	.1923
rs12899331	15	55,801,094	0.9294	.2307
rs10046	15	51,502,986	1.0332	.4888
rs1065778	15	51,520,206	1.0493	.3045
rs6564903	16	81,653,657	1.0189	.6982
rs11860694	16	84,457,447	1.0659	.1795
rs1299348	18	13,822,256	0.9914	.8618
rs11873029	18	46,617,055	0.9793	.7464
rs8094327	18	55,963,045	0.9682	.5946
rs12606138	18	55,993,944	0.9696	.6075
rs459962	21	15,963,120	1.0073	.9017
rs5965871	X	144,673,082	1.0753	.1997

Note *p*‐value is the unadjusted *p* value from genome‐wide association tests. Previous literature SNPs were included in genome‐wide association analyses, but may not have been in the top 100.

Abbreviation: Chr, chromosome.

## References

[brb31735-bib-0001] Becker, N. , Vasconcelos, M. , Oliveira, V. , Santos, F.C.D. , Bizarro, L. , Almeida, R.M.M.D. , … Carvalho, M.R.S. (2017). Genetic and environmental risk factors for developmental dyslexia in children: Systematic review of the last decade. Developmental Neuropsychology, 42(7–8), 423–445. 10.1080/87565641.2017.1374960 29068706

[brb31735-bib-0002] Beitchman, J.H. , & Young, A. (1997). Learning disorders with a special emphasis on reading disorders: A review of the past 10 years. Journal of the American Academy of Child and Adolescent Psychiatry, 36(8), 1020–1032. 10.1097/00004583-199708000-00009 9256582

[brb31735-bib-0003] Beneventi, H. , Tonnessen, F.E. , & Ersland, L. (2009). Dyslexic children show short‐term memory deficits in phonological storage and serial rehearsal: An fMRI study. International Journal of Neuroscience, 119(11), 2017–2043. 10.1080/00207450903139671 19863259

[brb31735-bib-0004] Berninger, V.W. , Abbott, R.D. , Vermeulen, K. , & Fulton, C.M. (2006). Paths to reading comprehension in at‐risk second‐grade readers. Journal of Learning Disabilities, 39(4), 334–351. 10.1177/00222194060390040701 16895158

[brb31735-bib-0005] Boyd, A. , Golding, J. , Macleod, J. , Lawlor, D.A. , Fraser, A. , Henderson, J. , … Davey Smith, G. (2013). Cohort Profile: The ‘Children of the 90s'—the index offspring of the Avon Longitudinal Study of Parents and Children. International Journal of Epidemiology, 42(1), 111–127. 10.1093/ije/dys064 22507743 PMC3600618

[brb31735-bib-0006] Braze, D. , Katz, L. , Magnuson, J.S. , Mencl, W.E. , Tabor, W. , Van Dyke, J.A. , … Shankweiler, D.P. (2016). Vocabulary does not complicate the simple view of reading. Reading and Writing, 29(3), 435–451. 10.1007/s11145-015-9608-6 26941478 PMC4761369

[brb31735-bib-0007] Bryant, P. , Nunes, T. , & Barros, R. (2014). The connection between children's knowledge and use of grapho‐phonic and morphemic units in written text and their learning at school. British Journal of Educational Psychology, 84(2), 211–225. 10.1111/bjep.12030 24251419

[brb31735-bib-0008] Carrion‐Castillo, A. , Franke, B. , & Fisher, S.E. (2013). Molecular genetics of dyslexia: An overview. Dyslexia, 19(4), 214–240. 10.1002/dys.1464 24133036

[brb31735-bib-0009] Catts, H.W. (2017). Early identification of reading disabilities In Cain K., Compton D., & Parrila R.K. (Eds.), Theories of reading development (pp. 311–332). Amsterdam, The Netherlands: John Benjamins Publishing.

[brb31735-bib-0010] Chang, C.C. , Chow, C.C. , Tellier, L.C.A.M. , Vattikuti, S. , Purcell, S.M. , & Lee, J.J. (2015). Second‐generation PLINK: Rising to the challenge of larger and richer datasets. GigaScience, 4(1), 1–16. 10.1186/s13742-015-0047-8 25722852 PMC4342193

[brb31735-bib-0011] Cho, S. , Kim, H. , Oh, S. , Kim, K. , & Park, T. (2009). Elastic‐net regularization approaches for genome‐wide association studies of rheumatoid arthritis. BMC Proceedings, 3(Suppl. 7), S25 10.1186/1753-6561-3-s7-s25 20018015 PMC2795922

[brb31735-bib-0012] Cirino, P.T. , Miciak, J. , Ahmed, Y. , Barnes, M.A. , Taylor, W.P. , & Gerst, E.H. (2018). Executive function: Association with multiple reading skills. Reading and Writing, 32(7), 1819–1846. 10.1007/s11145-018-9923-9 31680727 PMC6824553

[brb31735-bib-0013] Cope, N. , Eicher, J.D. , Meng, H. , Gibson, C.J. , Hager, K. , Lacadie, C. , … Gruen, J.R. (2012). Variants in the DYX2 locus are associated with altered brain activation in reading‐related brain regions in subjects with reading disability. NeuroImage, 63(1), 148–156. 10.1016/j.neuroimage.2012.06.037 22750057 PMC3518451

[brb31735-bib-0014] D'Mello, A.M. , & Gabrieli, J.D.E. (2018). Cognitive neuroscience of dyslexia. Language, Speech, and Hearing Services in Schools, 49(4), 798–809. 10.1044/2018_LSHSS-DYSLC-18-0020 30458541

[brb31735-bib-0015] Daniel, S.S. , Walsh, A.K. , Goldston, D.B. , Arnold, E.M. , Reboussin, B.A. , & Wood, F.B. (2006). Suicidality, school dropout, and reading problems among adolescents. Journal of Learning Disabilities, 39(6), 507–514. 10.1177/00222194060390060301 17165618

[brb31735-bib-0016] de Beer, J. , Engels, J. , Heerkens, Y. , & van der Klink, J. (2014). Factors influencing work participation of adults with developmental dyslexia: A systematic review. BMC Public Health, 14, 77 10.1186/1471-2458-14-77 24460949 PMC3913008

[brb31735-bib-0017] Deffenbacher, K.E. , Kenyon, J.B. , Hoover, D.M. , Olson, R.K. , Pennington, B.F. , DeFries, J.C. , & Smith, S.D. (2004). Refinement of the 6p21.3 quantitative trait locus influencing dyslexia: Linkage and association analyses. Human Genetics, 115(2), 128–138. 10.1007/s00439-004-1126-6 15138886

[brb31735-bib-0018] Devasenapathy, S. , Midha, R. , Naskar, T. , Mehta, A. , Prajapati, B. , Ummekulsum, M. , … Sinha, S. (2018). A pilot Indian family‐based association study between dyslexia and Reelin pathway genes, DCDC2 and ROBO1, identifies modest association with a triallelic unit TAT in the gene RELN. Asian Journal of Psychiatry, 37, 121–129. 10.1016/j.ajp.2018.08.020 30199849

[brb31735-bib-0019] Dollaghan, C.A. , Campbell, T.F. , Paradise, J.L. , Feldman, H.M. , Janosky, J.E. , Pitcairn, D.N. , & Kurs‐Lasky, M. (1999). Maternal education and measures of early speech and language. Journal of Speech, Language, and Hearing Research, 42(6), 1432–1443. 10.1044/jslhr.4206.1432 10599625

[brb31735-bib-0020] Durkin, M.E. , Avner, M.R. , Huh, C.G. , Yuan, B.Z. , Thorgeirsson, S.S. , & Popescu, N.C. (2005). DLC‐1, a Rho GTPase‐activating protein with tumor suppressor function, is essential for embryonic development. FEBS Letters, 579(5), 1191–1196. 10.1016/j.febslet.2004.12.090 15710412

[brb31735-bib-0021] Eicher, J.D. , Powers, N.R. , Miller, L.L. , Akshoomoff, N. , Amaral, D.G. , Bloss, C.S. , … Gruen, J.R. (2013). Genome‐wide association study of shared components of reading disability and language impairment. Genes, Brain, and Behavior, 12(8), 792–801. 10.1111/gbb.12085 PMC3904347 24024963

[brb31735-bib-0022] Eicher, J.D. , Powers, N.R. , Miller, L.L. , Mueller, K.L. , Mascheretti, S. , Marino, C. , … Gruen, J.R. (2014). Characterization of the DYX2 locus on chromosome 6p22 with reading disability, language impairment, and IQ. Human Genetics, 133(7), 869–881. 10.1007/s00439-014-1427-3 24509779 PMC4053598

[brb31735-bib-0023] Facoetti, A. , Gori, S. , Vicari, S. , & Menghini, D. (2019). Introduction to the special issue: Developmental dyslexia: From genes to remediation. Neuropsychologia, 130(June), 1–2. 10.1016/j.neuropsychologia.2019.06.003 31194982

[brb31735-bib-0024] Fan, J. , & Lv, J. (2008). Sure independene screening for ultrahigh dimensional feature space. Journal of the Royal Statistical Society: Series B (Statistical Methodology), 70(5), 849–911. 10.1111/j.1467-9868.2008.00674.x PMC2709408 19603084

[brb31735-bib-0025] Fisher, S.E. , Marlow, A.J. , Lamb, J. , Maestrini, E. , Williams, D.F. , Richardson, A.J. , … Monaco, A.P. (1999). A quantitative‐trait locus on chromosome 6p influences different aspects of developmental dyslexia. American Journal of Human Genetics, 64(1), 146–156. 10.1086/302190 9915953 PMC1377712

[brb31735-bib-0026] Folsom, T.D. , & Fatemi, S.H. (2013). The involvement of Reelin in neurodevelopmental disorders. Neuropharmacology, 68, 122–135. 10.1016/j.neuropharm.2012.08.015 22981949 PMC3632377

[brb31735-bib-0027] Fraser, A. , Macdonald‐Wallis, C. , Tilling, K. , Boyd, A. , Golding, J. , Davey Smith, G. , … Lawlor, D.A. (2013). Cohort profile: The Avon Longitudinal Study of Parents and Children: ALSPAC mothers cohort. International Journal of Epidemiology, 42(1), 97–110. 10.1093/ije/dys066 22507742 PMC3600619

[brb31735-bib-0028] Friend, A. , DeFries, J.C. , Wadsworth, S.J. , & Olson, R.K. (2007). Genetic and environmental influences on word recognition and spelling deficits as a function of age. Behavior Genetics, 37(3), 477–486. 10.1007/s10519-007-9145-4 17345157

[brb31735-bib-0029] Gathercole, S.E. , Willis, C.S. , Baddeley, A.D. , & Emslie, H. (1994). The children's test of nonword repetition: A test of phonological working memory. Memory, 2(2), 103–127. 10.1080/09658219408258940 7584287

[brb31735-bib-0030] Gayán, J. , & Olson, R.K. (2001). Genetic and environmental influences on orthographic and phonological skills in children with reading disabilities. Developmental Neuropsychology, 20(2), 483–507. 10.1207/S15326942DN2002_3 11892949

[brb31735-bib-0031] Gialluisi, A. , Guadalupe, T. , Francks, C. , & Fisher, S.E. (2017). Neuroimaging genetic analyses of novel candidate genes associated with reading and language. Brain and Language, 172, 9–15. 10.1016/j.bandl.2016.07.002 27476042

[brb31735-bib-0032] Gibson, C.J. , & Gruen, J.R. (2008). The human lexinome: Genes of language and reading. Journal of Communication Disorders, 41(5), 409–420. 10.1016/j.jcomdis.2008.03.003 18466916 PMC2488410

[brb31735-bib-0033] Gu, H. , Hou, F. , Liu, L. , Luo, X. , Nkomola, P.D. , Xie, X. , … Song, R. (2018). Genetic variants in the CNTNAP2 gene are associated with gender differences among dyslexic children in China. EBioMedicine, 34, 165–170. 10.1016/j.ebiom.2018.07.007 30017804 PMC6116347

[brb31735-bib-0034] Harding, J.F. , Morris, P.A. , & Hughes, D. (2015). The relationship between maternal education and children's academic outcomes: A theoretical framework. Journal of Marriage and Family, 77(1), 60–76. 10.1111/jomf.12156

[brb31735-bib-0035] Helland, T. , & Asbjornsen, A. (2003). Visual‐sequential and visuo‐spatial skills in dyslexia: Variations according to language comprehension and mathematics skills. Child Neuropsychology, 9(3), 208–220. 10.1076/chin.9.3.208.16456 13680410

[brb31735-bib-0036] Hofmeister, W. , Nilsson, D. , Topa, A. , Anderlid, B.‐M. , Darki, F. , Matsson, H. , … Lindstrand, A. (2015). CTNND2‐a candidate gene for reading problems and mild intellectual disability. Journal of Medical Genetics, 52(2), 111–122. 10.1136/jmedgenet-2014-102757 25473103

[brb31735-bib-0037] Hornyak, P. , Askwith, T. , Walker, S. , Komulainen, E. , Paradowski, M. , Pennicott, L.E. , … Oliver, A.W. (2016). Mode of action of DNA‐competitive small molecule inhibitors of tyrosyl DNA phosphodiesterase 2. The Biochemical Journal, 473(13), 1869–1879. 10.1042/BCJ20160180 27099339 PMC4925160

[brb31735-bib-0038] Ishii, K. , Kubo, K.I. , & Nakajima, K. (2016). Reelin and neuropsychiatric disorders. Frontiers in Cellular Neuroscience, 10, 229 10.3389/fncel.2016.00229 27803648 PMC5067484

[brb31735-bib-0039] Jerrim, J. , Vignoles, A. , Lingam, R. , & Friend, A. (2015). The socio‐economic gradient in children's reading skills and the role of genetics. British Educational Research Journal, 41(1), 6–29. 10.1002/berj.3143

[brb31735-bib-0040] Kamhi, A.G. , & Catts, H.W. (2012). Language and reading disabilities (3rd ed.). Boston, MA: Pearson.

[brb31735-bib-0041] Kershner, J.R. (2019). Neurobiological systems in dyslexia. Trends in Neuroscience and Education, 14, 11–24. 10.1016/j.tine.2018.12.001 30929855

[brb31735-bib-0042] Landi, N. , & Perdue, M.V. (2019). Neuroimaging genetics studies of specific reading disability and developmental language disorder: A review. Language and Linguistics Compass, 13(9), e12349 10.1111/lnc3.12349 31844423 PMC6913889

[brb31735-bib-0043] Language and Reading Research Consortium (2015). Learning to read: Should we keep things simple? Reading Research Quarterly, 50(2), 151–169. 10.1002/rrq.99

[brb31735-bib-0044] le Clercq, C.M.P. , van der Schroeff, M.P. , Rispens, J.E. , Ruytjens, L. , Goedegebure, A. , van Ingen, G. , & Franken, M.‐C. (2017). Shortened nonword repetition task (NWR‐S): A simple, quick, and less expensive outcome to identify children with combined specific language and reading impairment. Journal of Speech, Language, and Hearing Research, 60(8), 2241–2248. 10.1044/2017_JSLHR-L-16-0060 28702677

[brb31735-bib-0045] Lervåg, A. , Dolean, D. , Tincas, I. , & Melby‐Lervåg, M. (2019). Socioeconomic background, nonverbal IQ and school absence affects the development of vocabulary and reading comprehension in children living in severe poverty. Developmental Science, e12858 10.1111/desc.12858 31094030

[brb31735-bib-0046] Luciano, M. , Gow, A.J. , Pattie, A. , Bates, T.C. , & Deary, I.J. (2018). The influence of dyslexia candidate genes on reading skill in old age. Behavior Genetics, 48(5), 351–360. 10.1007/s10519-018-9913-3 29959602 PMC6097729

[brb31735-bib-0047] Lyytinen, H. , Ahonen, T. , Eklund, K.M. , Guttorm, T. , Kulju, P. , Laakso, M.‐L. , … Viholainen, H. (2004). Early development of children at familial risk for dyslexia ‐ follow‐up from birth to school age. Dyslexia, 10, 146–178. 10.1002/dys.274 15341196

[brb31735-bib-0048] Maeta, K. , Hattori, S. , Ikutomo, J. , Edamatsu, H. , Bilasy, S.E. , Miyakawa, T. , & Kataoka, T. (2018). Comprehensive behavioral analysis of mice deficient in Rapgef2 and Rapgef6, a subfamily of guanine nucleotide exchange factors for Rap small GTPases possessing the Ras/Rap‐associating domain. Molecular Brain, 11(1), 27 10.1186/s13041-018-0370-y 29747665 PMC5946393

[brb31735-bib-0049] Martin, A. , Kronbichler, M. , & Richlan, F. (2016). Dyslexic brain activation abnormalities in deep and shallow orthographies: A meta‐analysis of 28 functional neuroimaging studies. Human Brain Mapping, 37(7), 2676–2699. 10.1002/hbm.23202 27061464 PMC5103175

[brb31735-bib-0050] Martin, A. , Schurz, M. , Kronbichler, M. , & Richlan, F. (2015). Reading in the brain of children and adults: A meta‐analysis of 40 functional magnetic resonance imaging studies. Human Brain Mapping, 36(5), 1963–1981. 10.1002/hbm.22749 25628041 PMC4950303

[brb31735-bib-0051] Mascheretti, S. , Andreola, C. , Scaini, S. , & Sulpizio, S. (2018). Beyond genes: A systematic review of environmental risk factors in specific reading disorder. Research in Developmental Disabilities, 82(March), 147–152. 10.1016/j.ridd.2018.03.005 29566979

[brb31735-bib-0052] Mascheretti, S. , Bureau, A. , Battaglia, M. , Simone, D. , Quadrelli, E. , Croteau, J. , … Marino, C. (2013). An assessment of gene‐by‐environment interactions in developmental dyslexia‐related phenotypes. Genes, Brain, and Behavior, 12(1), 47–55. 10.1111/gbb.12000 23176554

[brb31735-bib-0053] Mascheretti, S. , Bureau, A. , Trezzi, V. , Giorda, R. , & Marino, C. (2015). An assessment of gene‐by‐gene interactions as a tool to unfold missing heritability in dyslexia. Human Genetics, 134(7), 749–760. 10.1007/s00439-015-1555-4 25916574

[brb31735-bib-0054] Mascheretti, S. , De Luca, A. , Trezzi, V. , Peruzzo, D. , Nordio, A. , Marino, C. , & Arrigoni, F. (2017). Neurogenetics of developmental dyslexia: From genes to behavior through brain neuroimaging and cognitive and sensorial mechanisms. Transl Psychiatry., 7(1), e987–e1015. 10.1038/tp.2016.240 28045463 PMC5545717

[brb31735-bib-0055] McCandliss, B.D. , & Noble, K.G. (2003). The development of reading impairment: A cognitive neuroscience model. Mental Retardation and Developmental Disabilities Research Reviews, 9(3), 196–204. 10.1002/mrdd.10080 12953299

[brb31735-bib-0056] Meng, H. , Smith, S.D. , Hager, K. , Held, M. , Liu, J. , Olson, R.K. , … Gruen, J.R. (2005). DCDC2 is associated with reading disability and modulates neuronal development in the brain. Proceedings of the National Academy of Sciences of the United States of America, 102(47), 17053–17058. 10.1073/pnas.0508591102 16278297 PMC1278934

[brb31735-bib-0057] Morken, F. , & Helland, T. (2013). Writing in dyslexia: Product and process. Dyslexia, 19(3), 131–148. 10.1002/dys.1455 23720272

[brb31735-bib-0058] Neale, M.D. , McKAY, M.F. , & Childs, G.H. (1986). The Neale analysis of reading ability – Revised. British Journal of Educational Psychology, 56(3), 346–356. 10.1111/j.2044-8279.1986.tb03047.x

[brb31735-bib-0059] Newbury, D.F. , Monaco, A.P. , & Paracchini, S. (2014). Reading and language disorders: The importance of both quantity and quality. Genes (Basel), 5(2), 285–309. 10.3390/genes5020285 24705331 PMC4094934

[brb31735-bib-0060] Niogi, S.N. , & McCandliss, B.D. (2006). Left lateralized white matter microstructure accounts for individual differences in reading ability and disability. Neuropsychologia, 44, 2178–2188. 10.1016/j.neuropsychologia.2006.01.011 16524602

[brb31735-bib-0061] Nunes, T. , Byrant, P. , & Olsson, J. (2003). Learning morphological and phonological spelling rules: An intervention study. Scientific Studies of Reading, 7(3), 289–307. 10.1207/S1532799XSSR0703

[brb31735-bib-0062] Paracchini, S. , Diaz, R. , & Stein, J. (2016). Advances in Dyslexia genetics—New insights into the role of brain asymmetries (Vol. 96). Cambridge, MA: Elsevier Ltd 10.1016/bs.adgen.2016.08.003 27968731

[brb31735-bib-0063] Paracchini, S. , Steer, C.D. , Buckingham, L.L. , Morris, A.P. , Ring, S. , Scerri, T. , … Monaco, A.P. (2008). Association of the KIAA0319 dyslexia susceptibility gene with reading skills in the general population. American Journal of Psychiatry, 165(12), 1576–1584. 10.1176/appi.ajp.2008.07121872 18829873

[brb31735-bib-0064] Peter, B. , Lancaster, H.S. , Vose, C. , Middleton, K. , & Stoel‐Gammon, C. (2017). Sequential processing deficit as a shared persisting biomarker in dyslexia and childhood apraxia of speech. Clinical Linguistics & Phonetics, 00(00), 1–31. 10.1080/02699206.2017.1375560 PMC6085870 28933620

[brb31735-bib-0065] Poelmans, G. , Buitelaar, J.K. , Pauls, D.L. , & Franke, B. (2011). A theoretical molecular network for dyslexia: Integrating available genetic findings. Molecular Psychiatry, 16(4), 365–382. 10.1038/mp.2010.105 20956978

[brb31735-bib-0066] Powers, N.R. , Eicher, J.D. , Miller, L.L. , Kong, Y. , Smith, S.D. , Pennington, B.F. , … Gruen, J.R. (2016). The regulatory element READ1 epistatically influences reading and language, with both deleterious and protective alleles. Journal of Medical Genetics, 53(3), 163–171. 10.1136/jmedgenet-2015-103418 26660103 PMC4789805

[brb31735-bib-0067] Price, K.M. , Wigg, K.G. , Feng, Y. , Blokland, K. , Wilkinson, M. , He, G. , … Barr, C.L. (2020). Genome‐Wide Association Study of Word Reading: Overlap with risk genes for neurodevelopmental disorders. Genes, Brain, and Behavior, e12648 10.1111/gbb.12648 32108986

[brb31735-bib-0068] Rabinowitz, J.A. , Kuo, S.I.C. , Felder, W. , Musci, R.J. , Bettencourt, A. , Benke, K. , … Ialongo, N.S. (2019). Associations between an educational attainment polygenic score with educational attainment in an African American sample. Genes, Brain, and Behavior, 18(5), 1–10. 10.1111/gbb.12558 PMC7008934 30793481

[brb31735-bib-0069] Reimand, J. , Arak, T. , Adler, P. , Kolberg, L. , Reisberg, S. , Peterson, H. , & Vilo, J. (2016). g:Profiler‐a web server for functional interpretation of gene lists (2016 update). Nucleic Acids Research, 44(W1), W83–W89. 10.1093/nar/gkw199 27098042 PMC4987867

[brb31735-bib-0070] Rendall, A.R. , Perrino, P.A. , LoTurco, J.J. , & Fitch, R.H. (2019). Evaluation of visual motion perception ability in mice with knockout of the dyslexia candidate susceptibility gene Dcdc2. Genes, Brain, and Behavior, 18(5), 1–8. 10.1111/gbb.12450 29232042

[brb31735-bib-0071] Rosner, J. , & Simon, D.P. (1971). The auditory analysis test. Journal of Learning Disabilities, 4(7), 384–392. 10.1177/002221947100400706

[brb31735-bib-0072] Rust, J. (1996). Weschler objective language dimensions manual. London, UK: The Psychological Corporation.

[brb31735-bib-0073] Rust, J. , Golombok, S. , & Trickey, G. (1993). WORD. Wechsler objective reading dimensions. London, UK: Psychological Corp.

[brb31735-bib-0074] Scerri, T.S. , Darki, F. , Newbury, D.F. , Whitehouse, A.J.O. , Peyrard‐Janvid, M. , Matsson, H. , … Paracchini, S. (2012). The dyslexia candidate locus on 2p12 is associated with general cognitive ability and white matter structure. PLoS One, 7(11), e50321 10.1371/journal.pone.0050321 23209710 PMC3509064

[brb31735-bib-0075] Scerri, T.S. , Morris, A.P. , Buckingham, L.L. , Newbury, D.F. , Miller, L.L. , Monaco, A.P. , … Paracchini, S. (2011). DCDC2, KIAA0319 and CMIP are associated with reading‐related traits. Biological Psychiatry, 70(3), 237–245. 10.1016/j.biopsych.2011.02.005 21457949 PMC3139836

[brb31735-bib-0076] Shannon, P. , Markiel, A. , Ozier, O. , Baliga, N.S. , Wang, J.T. , Ramag, D. , … Ideker, T. (2003). Cytoscape: A software Environment for integrated models of biomolecular interaction networks. Genome Research, 13(11), 2498–2504. 10.1101/gr.1239303 14597658 PMC403769

[brb31735-bib-0078] Shao, S. , Niu, Y. , Zhang, X. , Kong, R. , Wang, J. , Liu, L. , … Song, R. (2016). Opposite associations between individual KIAA0319 polymorphisms and developmental dyslexia risk across populations: A stratified meta‐analysis by the study population. Scientific Reports, 6(1). 10.1038/srep30454 PMC4964335 27464509

[brb31735-bib-0079] Sharma, M. , Purdy, S.C. , & Kelly, A.S. (2009). Comorbidity of auditory processing, language, and reading disorders. Journal of Speech, Language, and Hearing Research, 52(3), 706–722. 10.1044/1092-4388(2008/07-0226) 19064904

[brb31735-bib-0080] Skeide, M.A. , Kirsten, H. , Kraft, I. , Schaadt, G. , Müller, B. , Neef, N. , … Friederici, A.D. (2015). Genetic dyslexia risk variant is related to neural connectivity patterns underlying phonological awareness in children. NeuroImage, 118, 414–421. 10.1016/j.neuroimage.2015.06.024 26080313

[brb31735-bib-0081] Sperling, A.J. , Lu, Z.L. , Manis, F.R. , & Seidenberg, M.S. (2005). Deficits in perceptual noise exclusion in developmental dyslexia. Nature Neuroscience, 8(7), 862–863. 10.1038/nn1474 15924138

[brb31735-bib-0082] Stein, J.L. , Medland, S.E. , Vasquez, A.A. , Hibar, D.P. , Senstad, R.E. , Winkler, A.M. , … Thompson, P.M. (2012). Identification of common variants associated with human hippocampal and intracranial volumes. Nature Genetics, 44(5), 552–561. 10.1038/ng.2250 22504417 PMC3635491

[brb31735-bib-0083] Stojanovik, V. , & Riddell, P. (2008). Expressive versus receptive language skills in specific reading disorder. Clinical Linguistics & Phonetics, 22(4‐5), 305–310. 10.1080/02699200801919349 18415729

[brb31735-bib-0084] Tai, Y.K. , Healy, K.D. , Der, C.J. , Sciaky, N. , Bang, Y.J. , & Juliano, R.L. (2008). Effects of structure of Rho GTPase‐activating protein DLC‐1 on cell morphology and migration. Journal of Biological Chemistry, 283(47), 32762–32770. 10.1074/jbc.M800617200 18786931 PMC2583296

[brb31735-bib-0085] Tomblin, J.B. , Zhang, X. , Buckwalter, P. , & Catts, H.W. (2000). The association of reading disability, behavioral disorders, and language impairment among second‐grade children. Journal of Child Psychology and Psychiatry and Allied Disciplines, 41(4), 473–482. 10.1017/S002196300000559X 10836677

[brb31735-bib-0086] Torppa, M. , Lyytinen, P. , Erskine, J. , Eklund, K.M. , & Lyytinen, H. (2010). Language development, literacy skills, and predictive connections to reading in Finnish children with and without familial risk for Dyslexia. Journal of Learning Disabilities, 43(4), 308–321. 10.1177/0022219410369096 20479461

[brb31735-bib-0087] Tunmer, W.E. , & Chapman, J.W. (2012). The Simple View of Reading Redux: Vocabulary knowledge and the independent components hypothesis. Journal of Learning Disabilities, 45(5), 453–466. 10.1177/0022219411432685 22293683

[brb31735-bib-0088] Van Der Leij, A. , Van Bergen, E. , Van Zuijen, T. , de Jong, P.F. , Maurits, N.M. , & Maassen, B. (2013). Precursors of developmental dyslexia: An overview of the longitudinal Dutch dyslexia programme study. Dyslexia, 19(4), 191–213. 10.1002/dys.1463 24133035

[brb31735-bib-0089] Wadsworth, S.J. , DeFries, J.C. , Olson, R.K. , & Willcutt, E.G. (2007). Colorado longitudinal twin study of reading disability. Ann Dyslexia., 57(2), 139–160. 10.1007/s11881-007-0009-7 18060583

[brb31735-bib-0090] Waldie, K.E. , Wilson, A.J. , Reece, R. , Moreau, D. , Roberts, R. , & Moreau, D. (2017). Reading network in dyslexia: Similar, yet different. Brain and Language, 174, 29–41. 10.1016/j.bandl.2017.07.004 28715717

[brb31735-bib-0091] Waldmann, P. , Mészáros, G. , Gredler, B. , Fuerst, C. , & Sölkner, J. (2013). Evaluation of the lasso and the elastic net in genome‐wide association studies. Frontiers in Genetics, 4, 1–11. 10.3389/fgene.2013.00270 PMC3850240 24363662

[brb31735-bib-0092] Wechsler, D. , Golombok, S. , & Rust, J. (1992). WISC‐III UK Wechsler intelligence scale for children: UK manual. Sidcup, UK: The Psychological Corporation.

[brb31735-bib-0093] Willcutt, E.G. , Pennington, B.F. , Duncan, L. , Smith, S.D. , Keenan, J.M. , Wadsworth, S. , … Olson, R.K. (2011). Understanding the complex etiologies of developmental disorders: Behavioral and molecular genetic approaches. Journal of Developmental and Behavioral Pediatrics, 31(7), 533–544. 10.1097/DBP.0b013e3181ef42a1.Understanding PMC2953861 20814254

[brb31735-bib-0094] Zhao, H. , Chen, Y. , Zhang, B. , & Zuo, P.X. (2016). KIAA0319 gene polymorphisms are associated with developmental dyslexia in Chinese Uyghur children. Journal of Human Genetics, 61(8), 745–752. 10.1038/jhg.2016.40 27098879 PMC4999827

[brb31735-bib-0095] Zou, H. (2006). The adaptive lasso and its oracle properties. Journal of American Statistical Association, 101(476), 1418–1429. 10.1198/016214506000000735

